# Supplementary material for: Genome-Wide Associations and Confirmatory Meta-Analyses in Diabetic Retinopathy
Source: Genes (Basel). 2023 Mar 5;14(3):653. doi: 10.3390/genes14030653 (PMC10048213; doi:10.3390/genes14030653)
Supplement: Supplementary file 1 [file genes-14-00653-s001.zip › genes-2223016-supplementary.pdf]

Genome-Wide Associations and Confirmatory Meta-Analyses in Diabetic Retinopathy

Xinting Yu [1,†] and Shisong Rong [2,*,†]

[1] Department of Medicine, Brigham and Women's Hospital, Mass General Brigham, Harvard Medical School, Boston, MA 02445, USA

[2] Department of Ophthalmology, Massachusetts Eye and Ear, Mass General Brigham, Harvard Medical School, Boston, MA 02445, USA

* Correspondence: shisong_rong@meei.harvard.edu

† These authors contributed equally to this work.

Supplementary materials:

Table S1. Search strategies for identification of genome-wide association study of diabetic retinopathy in PubMed/MEDLINE

Table S2. Search strategies for genetic association studies of top genome-wide association study signals of diabetic retinopathy

Table S3. Summary of genome-wide significant gene loci in diabetic retinopathy

Table S4. Summary of genome-wide significant loci in proliferative diabetic retinopathy, severe diabetic retinopathy, and vision-threatening diabetic retinopathy

Table S5. Summary of genome-wide significant loci in diabetic macular edema/diabetic maculopathy

Table S6. Quality assessments of included case-control studies

Figure S1. GWAS catalog and literature search and results of literature review

Figure S2. Literature search for replication studies of top GWAS signals

Figure S3. Funnel plots of studies outcomes used in meta-analysis

Figure S4. Meta-analysis of genetic association of rs7903146 (T, TCF7L2) with diabetic retinopathy

Table S1. Search strategies for identification of genome-wide association study of diabetic retinopathy in PubMed/MEDLINE

Search number	Query
1	Diabetic Retinopathy [MeSH]
2	(Diabetes Mellitus [MeSH]) OR (Diabet* [tiab])
3	Diabet* [tiab]
4	Diabetes Mellitus [MeSH]
5	((((((Edema [tiab]) OR (Oedema [tiab])) AND (Macular [tiab])) OR (Maculopath* [tiab])) OR (Retinopath* [tiab])) OR (Microaneurysm* [tiab])) OR (Neovascular* [tiab])
6	Neovascular* [tiab]
7	Microaneurysm* [tiab]
8	Retinopath* [tiab]
9	Maculopath* [tiab]
10	((Edema [tiab]) OR (Oedema [tiab])) AND (Macular [tiab])
11	Macular [tiab]
12	Oedema [tiab]
13	Edema [tiab]
14	1 OR 2 OR 3 OR 4 OR 5 OR 6 OR 7 OR 8 OR 9 OR 10 OR 11 OR 12 OR 13
15	((genome-wide association studies) OR (genome wide association studies)) OR (GWAS)
16	(Medical Genetics OR genotype OR genetics[Subheading] AND genetics)
17	14 AND 15 AND 16

Table S2. Search strategies for genetic association studies of top genome-wide association study signals of diabetic retinopathy

Search number	Query
**Genetic studies of top gene loci identified in DR GWASs**	
1	(rs17376456 OR rs2038823 OR rs12630354 OR rs4838605 OR rs12219125 OR rs202069793 OR rs4462262)
2	(KIAA0825 OR HS6ST3 OR THRAP3P1 OR STT3B OR ARHGAP22 OR AMD1P1 OR PLXDC2 OR OR13D3P OR OR13D1)
3	(Medical Genetics OR genotype OR genetics[Subheading] AND genetics)
4	"diabetic retinopathy"[MeSH Terms] OR ("diabetic"[All Fields] AND "retinopathy"[All Fields]) OR "diabetic retinopathy"[All Fields]
5	((1 OR 2) AND 3) AND 4
**Genetic studies of top gene loci identified in PDR GWASs**	
1	rs3081219 OR rs3913535 OR rs11018670 OR rs72740408 OR rs184340784 OR rs1065386 OR rs4726066 OR rs200295620
2	WDR72 OR NOX4 OR FOLH1B OR HNRNPA1P46 OR LINC01646 OR HLA-B OR PRKAG2 OR GOLIM4 OR EGFEM1P
3	(Medical Genetics OR genotype OR genetics[Subheading] AND genetics)
4	proliferative diabetic retinopathy
5	((1 OR 2) AND 3) AND 4

Table S3. Summary of genome-wide significant gene loci in diabetic retinopathy

	SNP	Chr: Position (hg19)	Effect allele	Discovery population	Replication population	Discovery N	EAF	OR / Beta	95% CI	P value	Mapped gene	Reference
1	rs17376456	Chr5:93557702	A	Chinese	None	749*	NR	NR	NR	3.00E-15	KIAA0825/C5orf36	[13]
2	rs2038823	Chr13:96951433	C	Chinese	None	749*	NR	NR	NR	5.00E-11	HS6ST3	[13]
3	rs12630354	Chr3:31501119	T	Japanese	Japanese	8880	0.411	1.16	[1.11-1.22]	7.00E-10	THRAP3P1, STT3B	[17]
4	rs4838605	Chr10:49699957	C	Chinese	None	749*	NR	NR	NR	2.00E-09	ARHGAP22	[13]
5	rs12219125	Chr10:20593087	T	Chinese	None	749*	NR	NR	NR	9.00E-09	AMD1P1, PLXDC2	[13]
6	rs202069793	Chr9:107475295	NR	African American, Afro-Caribbean, European	Asian, European, Hispanic	5857	NR	1.39	NR	6.00E-08	OR13D3P, OR13D1	[26]
7	rs4462262	Chr10:59189178	C	Chinese	None	749*	NR	NR	NR	9.00E-08	ZWINT-MRPS35P3	[13]
8	rs2104455	Chr6:9368518	NR	African American, Afro-Caribbean, European	Asian, European, Hispanic	5857	NR	1.57	NR	1.00E-07	HULC	[26]
9	rs476141	Chr1:244176424	A	European	None	2829	0.51	1.37	NR	1.00E-07	LINC02774	[21]
10	rs75360147	Chr9:86964688	NR	African American, Afro-Caribbean, European	Asian, European, Hispanic	5857	NR	2.17	NR	2.00E-07	SLC28A3	[26]
11	rs7861760	Chr9:34368674	G	Japanese	Japanese	8880	0.65	1.14	[1.09-1.20]	2.00E-07	MYORG	[17]
12	rs74152685	Chr1:247811291	NR	African American, Afro-Caribbean, European	Asian, European, Hispanic	5857	NR	2.564103	NR	3.00E-07	OR3D1P, OR14L1P	[26]
13	rs1571942	Chr10:20542634	C	Chinese	None	749*	NR	NR	NR	3.00E-07	Unknown	[13]
14	rs2811893	Chr1:59162148	T	Chinese	None	749*	NR	NR	NR	3.00E-07	MYSM1	[13]
15	rs140508424	Chr9:112695256	C	Japanese	Japanese	8880	0.985	1.53	[1.30-1.81]	3.00E-07	PALM2AKAP2	[17]
16	rs4470583	Chr4:162250932	A	Chinese	None	749*	NR	NR	NR	4.00E-07	TBC1D19, FSTL5	[13]
17	rs2471299	Chr7:139982756	A	Japanese	Japanese	8880	0.53	1.13	[1.08-1.18]	4.00E-07	PPP1R2P6, RNU1-58P	[17]

	SNP	Chr: Position (hg19)	Effect allele	Discovery population	Replication population	Discovery N	EAF	OR / Beta	95% CI	P value	Mapped gene	Reference
18	rs2300993	Chr5:109038629	NR	African American, Afro-Caribbean, European	Asian, European, Hispanic	5857	NR	1.83	NR	5.00E-07	MAN2A1	[26]
19	rs184340784	Chr1:4589883	NR	African American, Afro-Caribbean, European	Asian, European, Hispanic	5857	NR	NR	NR	6.00E-07	LINC01646	[26]
20	rs148995025	Chr5:7085207	NR	African American, Afro-Caribbean, European	Asian, European, Hispanic	5857	NR	3.41	NR	6.00E-07	LINC02196	[26]
21	rs4787008	Chr16:7415552	G	European	None	2829	0.17	1.47	NR	6.00E-07	RBFOX1	[21]
22	rs13064954	Chr3:156854742	G	European	None	2829	0.04	1.02	NR	7.00E-07	CCNL1, LINC00881	[21]
23	rs12656571	Chr5:109078040	NR	African American, Afro-Caribbean, European	Asian, European, Hispanic	5857	NR	1.89	NR	8.00E-07	MAN2A1	[26]
24	rs9866141	Chr3:156950579	T	European	None	2829	0.04	1.02	NR	9.00E-07	RNA5SP146, VEPH1	[21]
25	rs9362054	Chr6:85178268	T	Japanese	Asian	446	0.291	1.4	NR	1.00E-06	LINC01611	[14]
26	rs114790220	Chr8:5163956	NR	African American, Afro-Caribbean, European	Asian, European, Hispanic	5857	NR	2.9411764	NR	1.00E-06	Unknown	[26]
27	rs11771617	Chr7:125373426	NR	African American, Afro-Caribbean, European	Asian, European, Hispanic	5857	NR	3.12	NR	1.00E-06	Unknown	[26]
28	rs11662496	Chr18:41275397	NR	African American, Afro-Caribbean, European	Asian, European, Hispanic	5857	NR	1.3888888	NR	1.00E-06	Unknown	[26]

	SNP	Chr: Position (hg19)	Effect allele	Discovery population	Replication population	Discovery N	EAF	OR / Beta	95% CI	P value	Mapped gene	Reference
29	rs142610219	Chr8:116918264	NR	African American, Afro-Caribbean, European	Asian, European, Hispanic	5857	NR	NR	NR	1.00E-06	LINC00536, TRPS1	[26]
30	rs200295620	Chr3:167890186	NR	African American, Afro-Caribbean, European	Asian, European, Hispanic	5857	NR	2.42	NR	1.00E-06	GOLIM4, EGFEM1P	[26]
31	rs61741249	Chr7:157309591	NR	African American, Afro-Caribbean, European	Asian, European, Hispanic	5857	NR	6.25	NR	1.00E-06	PTPRN2, DNAJB6	[26]
32	rs17404956	Chr5:166389493	A	European	None	2829	0.9	1.16	NR	1.00E-06	LINC01947	[21]
33	rs115634195	Chr10:25735812	NR	African American, Afro-Caribbean, European	Asian, European, Hispanic	5857	NR	6.25	NR	2.00E-06	GPR158	[26]
34	rs9882204	Chr3:167566283	NR	African American, Afro-Caribbean, European	Asian, European, Hispanic	5857	NR	1.32	NR	2.00E-06	LRRC77P, SERPINI1	[26]
35	rs184033309	Chr5:103622556	NR	African American, Afro-Caribbean, European	Asian, European, Hispanic	5857	NR	3.030303	NR	2.00E-06	LINC02163	[26]
36	rs145764941	Chr5:67857981	NR	African American, Afro-Caribbean, European	Asian, European, Hispanic	5857	NR	1.63	NR	2.00E-06	Unknown	[26]
37	rs4901258	Chr14:52696282	NR	African American, Afro-Caribbean, European	Asian, European, Hispanic	5857	NR	1.44	NR	2.00E-06	LINC02319, PTGDR	[26]

	SNP	Chr: Position (hg19)	Effect allele	Discovery population	Replication population	Discovery N	EAF	OR / Beta	95% CI	P value	Mapped gene	Reference
38	rs201581084	Chr1:158733766	NR	African American, Afro-Caribbean, European	Asian, European, Hispanic	5857	NR	1.44	NR	2.00E-06	OR6N1	[26]
39	rs115489684	Chr5:142116464	NR	African American, Afro-Caribbean, European	Asian, European, Hispanic	5857	NR	1.754386	NR	2.00E-06	LINC01844	[26]
40	rs190634129	Chr4:143341715	NR	African American, Afro-Caribbean, European	Asian, European, Hispanic	5857	NR	3.448276	NR	2.00E-06	INPP4B	[26]
41	rs73194819	Chr2:13252874	NR	African American, Afro-Caribbean, European	Asian, European, Hispanic	5857	NR	1.5384616	NR	2.00E-06	Unknown	[26]
42	rs202189921	Chr2:236562109	NR	African American, Afro-Caribbean, European	Asian, European, Hispanic	5857	NR	3.73	NR	2.00E-06	AGAP1	[26]
43	rs7139352	Chr12:61345749	NR	African American, Afro-Caribbean, European	Asian, European, Hispanic	5857	NR	1.46	NR	2.00E-06	Unknown	[26]
44	rs201685870	Chr5:98692372	NR	African American, Afro-Caribbean, European	Asian, European, Hispanic	5857	NR	1.4492754	NR	2.00E-06	ADAMTS6	[26]
45	rs74816310	Chr5:73266734	NR	African American, Afro-Caribbean, European	Asian, European, Hispanic	5857	NR	NR	NR	2.00E-06	ARHGEF28	[26]

	SNP	Chr: Position (hg19)	Effect allele	Discovery population	Replication population	Discovery N	EAF	OR / Beta	95% CI	P value	Mapped gene	Reference
46	rs74966374	Chr5:55905540	NR	African American, Afro-Caribbean, European	Asian, European, Hispanic	5857	NR	2.13	NR	2.00E-06	C5orf67	[26]
47	rs10878791	Chr12:68609046	NR	African American, Afro-Caribbean, European	Asian, European, Hispanic	5857	NR	1.4925373	NR	2.00E-06	IFNG-AS1, IL26	[26]
48	rs115885880	Chr10:105720104	NR	African American, Afro-Caribbean, European	Asian, European, Hispanic	5857	NR	10	NR	2.00E-06	SLK, STN1	[26]
49	rs1360935	Chr13:55143426	NR	African American, Afro-Caribbean, European	Asian, European, Hispanic	5857	NR	3.7037036	NR	2.00E-06	Unknown	[26]
50	rs10927101	Chr1:244173872	A	European	None	2829	0.38	1.33	NR	2.00E-06	LINC02774	[21]
51	rs10403021	Chr19:30079604	C	European	None	2829	0.66	1.01	NR	2.00E-06	VSTM2B, POP4	[21]
52	rs1074390	Chr15:80080307	G	Japanese	Japanese	8880	0.412	1.12	[1.07-1.17]	2.00E-06	RNU6-667P	[17]
53	rs1559674	Chr15:62106284	NR	African American, Afro-Caribbean, European	Asian, European, Hispanic	5857	NR	1.4084507	NR	3.00E-06	VPS13C, LINC02349	[26]
54	rs12095420	Chr1:226788952	NR	African American, Afro-Caribbean, European	Asian, European, Hispanic	5857	NR	7.04	NR	3.00E-06	STUM	[26]
55	rs150557761	Chr7:63671293	NR	African American, Afro-Caribbean, European	Asian, European, Hispanic	5857	NR	2.0833335	NR	3.00E-06	ZNF735	[26]

	SNP	Chr: Position (hg19)	Effect allele	Discovery population	Replication population	Discovery N	EAF	OR / Beta	95% CI	P value	Mapped gene	Reference
56	rs7332766	Chr13:106007555	NR	African American, Afro-Caribbean, European	Asian, European, Hispanic	5857	NR	1.4925373	NR	3.00E-06	Unknown	[26]
57	rs2696835	Chr16:86365571	C	European	None	2829	0.03	2.27	NR	3.00E-06	LINC00917	[21]
58	rs7772697	Chr6:149435111	T	European	None	2829	0.58	1.35	NR	3.00E-06	UST	[21]
59	rs1970671	Chr18:52863108	G	European	None	2829	0.29	1.37	NR	3.00E-06	RNA5SP459, TCF4	[21]
60	rs2115386	Chr19:7196565	C	European	None	2829	0.55	1.12	NR	3.00E-06	INSR	[21]
61	rs10199521	Chr2:2519513	T	European	None	2829	0.22	1.46	NR	3.00E-06	Unknown	[21]
62	rs1894151	Chr18:53763451	G	Chinese	None	412	NR	3.96	[2.23-7.03]	3.00E-06	LINC01905, LINC01539	[16]
63	rs145279814	Chr1:72796513	NR	African American, Afro-Caribbean, European	Asian, European, Hispanic	5857	NR	2.78	NR	4.00E-06	RPL31P12	[26]
64	rs6763376	Chr3:167604119	NR	African American, Afro-Caribbean, European	Asian, European, Hispanic	5857	NR	1.57	NR	4.00E-06	LRRC77P	[26]
65	rs78141810	Chr18:35931012	NR	African American, Afro-Caribbean, European	Asian, European, Hispanic	5857	NR	2.2727273	NR	4.00E-06	Unknown	[26]
66	rs6702784	Chr1:36904720	C	European	None	2829	0.07	1.08	NR	4.00E-06	OSCP1	[21]
67	rs1342038	Chr1:173301516	G	European	None	2829	0.64	1.49	NR	4.00E-06	PRDX6-AS1	[21]
68	rs34766496	Chr4:35996710	A	Chinese	None	412	NR	2.3	[1.61-3.29]	4.00E-06	ARAP2	[16]
69	rs62328468	Chr4:156735073	T	Chinese	None	412	NR	2.21	[1.58-3.09]	4.00E-06	ASIC5, GUCY1B1	[16]
70	rs3007729	Chr1:18795255	T	European	None	2829	0.65	1.35	NR	5.00E-06	IGSF21, KLHDC7A	[21]
71	rs74305293	Chr8:83575369	T	Japanese	Japanese	8880	0.279	1.14	[1.08-1.20]	5.00E-06	Unknown	[17]
72	rs10910200	Chr1:233643856	G	European	None	2829	0.25	1.35	NR	6.00E-06	RNU4-77P	[21]

	SNP	Chr: Position (hg19)	Effect allele	Discovery population	Replication population	Discovery N	EAF	OR / Beta	95% CI	P value	Mapped gene	Reference
73	rs11765845	Chr7:28391142	A	European	None	2829	0.29	1.02	NR	7.00E-06	CREB5	[21]
74	rs11867934	Chr17:16933404	C	European	None	2829	0.79	1.43	NR	7.00E-06	MPRIP, LINC02090	[21]
75	rs10432638	Chr2:24202512	NR	African American, Afro-Caribbean, European	Asian, European, Hispanic	5857	NR	1.1235955	NR	8.00E-06	UBXN2A	[26]
76	rs1073203	Chr5:125319456	G	European	None	2829	0.13	1.54	NR	9.00E-06	RPSAP37	[21]

Chr, chromosome; CI, confidence interval; EAF, effect allele frequency; NR, not reported; OR, odds ratio; SNP, single nucleotide polymorphism

Table S4. Summary of genome-wide significant loci in proliferative diabetic retinopathy, severe diabetic retinopathy, and vision-threatening diabetic retinopathy

	SNP	Chr:position (hg19)	Effect allele	Discovery population	Replication population	Discovery N	EAF	OR / Beta	95% CI	P value	Mapped gene	Reference
1	rs3081219	Chr15:53876170	C	African	African American or Afro-Caribbean	291	0.13	1.29	[1.19-1.41]	1.00E-09	WDR72	[28]
2	rs3913535	Chr11:89096757	C	Scottish (European)	Independent meta-analysis	4666	0.411	1.55	[1.34-1.79]	4.00E-09	NOX4	[25]
3	rs11018670	Chr11:89356628	G	Scottish (European)	Independent meta-analysis	4666	0.427	1.55	[1.33-1.80]	1.00E-08	FOLH1B	[25]
4	rs72740408	Chr1:191105831	A	African	African American or Afro-Caribbean	291	0.03	1.52	[1.31-1.76]	2.00E-08	HNRNPA1P46	[28]
5	rs184340784	Chr1:4589883	NR	African American, Afro-Caribbean, European	Asian, European, Hispanic	5857	NR	NR	NR	4.00E-08	LINC01646	[26]
6	rs1065386	Chr6:31324547	C	African	African American or Afro-Caribbean	291	0.43	1.17	[1.11-1.24]	5.00E-08	HLA-B	[28]
7	rs4726066	Chr7:151322272	NR	African American, Afro-Caribbean, European	Asian, European, Hispanic	5857	NR	NR	NR	5.00E-08	PRKAG2	[26]
8	rs200295620	Chr3:167890186	NR	African American, Afro-Caribbean, European	Asian, European, Hispanic	5857	NR	1.39	NR	7.00E-08	GOLIM4-EGFEM1P	[26]
9	rs78464534	Chr2:115991851	NR	African American, Afro-Caribbean, European	Asian, European, Hispanic	5857	NR	NR	NR	3.00E-07	DPP10	[26]

	SNP	Chr:position (hg19)	Effect allele	Discovery population	Replication population	Discovery N	EAF	OR / Beta	95% CI	P value	Mapped gene	Reference
10	rs114921230	Chr1:226782862	NR	African American, Afro-Caribbean, European	Asian, European, Hispanic	5857	NR	1.9	NR	5.00E-07	STUM	[26]
11	rs1000708	Chr12:60083488	NR	African American, Afro-Caribbean, European	Asian, European, Hispanic	5857	NR	NR	NR	7.00E-07	SLC16A7	[26]
12	rs74161190	Chr10:132535794	NR	African American, Afro-Caribbean, European	Asian, European, Hispanic	5857	NR	3.125	NR	7.00E-07	Unknown	[26]
13	rs9896052	Chr17:73418862	A	Australians (Whites)	European, Indian	844	0.294	1.67	[1.30-2.15]	7.00E-07	Y_RNA-RNU6-938P	[23]
14	rs71354195	Chr19:36876318	NR	African American, Afro-Caribbean, European	Asian, European, Hispanic	5857	NR	2.42	NR	8.00E-07	ZFP82	[26]
15	rs116396065	Chr8:122129270	NR	African American, Afro-Caribbean, European	Asian, European, Hispanic	5857	NR	NR	NR	1.00E-06	Unknown	[26]
16	rs201584991	Chr8:125991725	NR	African American, Afro-Caribbean, European	Asian, European, Hispanic	5857	NR	1.63	NR	1.00E-06	ZNF572-SQLE	[26]
17	rs74705672	Chr8:133054627	NR	African American, Afro-Caribbean, European	Asian, European, Hispanic	5857	NR	1.4492754	NR	1.00E-06	OC90	[26]
18	rs2064196	Chr6:144587183	NR	African American, Afro-Caribbean, European	Asian, European, Hispanic	5857	NR	1.8181818	NR	1.00E-06	TPT1P4-UTRN	[26]

	SNP	Chr:position (hg19)	Effect allele	Discovery population	Replication population	Discovery N	EAF	OR / Beta	95% CI	P value	Mapped gene	Reference
19	rs61811867	Chr1:154775244	NR	African American, Afro-Caribbean, European	Asian, European, Hispanic	5857	NR	NR	NR	1.00E-06	KCNN3	[26]
20	rs200197449	Chr14:101156146	NR	African American, Afro-Caribbean, European	Asian, European, Hispanic	5857	NR	1.44	NR	2.00E-06	DLK1-LINC00523	[26]
21	rs11575234	Chr12:56744276	NR	African American, Afro-Caribbean, European	Asian, European, Hispanic	5857	NR	1.754386	NR	2.00E-06	STAT2	[26]
22	rs12447665	Chr16:5537598	NR	African American, Afro-Caribbean, European	Asian, European, Hispanic	5857	NR	1.83	NR	2.00E-06	RBFOX1	[26]
23	rs1566115	Chr6:99632323	NR	African American, Afro-Caribbean, European	Asian, European, Hispanic	5857	NR	1.87	NR	2.00E-06	BDH2P1-FAXC	[26]
24	rs4129798	Chr3:18280998	NR	African American, Afro-Caribbean, European	Asian, European, Hispanic	5857	NR	1.8181818	NR	2.00E-06	TBC1D5	[26]
25	rs9446832	Chr6:73783508	NR	African American, Afro-Caribbean, European	Asian, European, Hispanic	5857	NR	NR	NR	2.00E-06	KCNQ5	[26]
26	rs10560003	Chr3:115378466	T	African	African American or Afro-Caribbean	291	0.02	1.52	[1.28-1.81]	3.00E-06	GAP43	[28]
27	rs918519	Chr5:158826357	NR	Australians (European)	None	881	0.769	2.86	[1.85-4.55]	4.00E-06	LINC01845-IL12B	[24]

	SNP	Chr:position (hg19)	Effect allele	Discovery population	Replication population	Discovery N	EAF	OR / Beta	95% CI	P value	Mapped gene	Reference
28	rs1158314	Chr14:80427732	G	Australians (European)	None	881	0.4	2.16	[1.56-3.00]	4.00E-06	NRXN3	[24]
29	rs9543976	Chr13:76136648	G	Chinese	Hispanic or Latin American	1007	0.3	1.6	NR	7.00E-06	UCHL3	[22]

Chr, chromosome; CI, confidence interval; EAF, effect allele frequency; NR, not reported; OR, odds ratio; SNP, single nucleotide polymorphism

Table S5. Summary of genome-wide significant loci in diabetic macular edema/diabetic maculopathy

	SNP	Chr:position	Effect allele	Discovery population	Replication population	Discovery N	EAF	OR / Beta	95% CI	P value	Mapped gene	Reference
1	rs9966620	Chr18:21680735	A	European	None	1843	0.0964	1.95	[1.71-2.19]	7.00E-08	TTC39C	[27]
2	rs3818329	Chr1:192627760	NR	European	None	1843	NR	1.87	[1.474-2.361]	2.00E-07	RGS13	[27]
3	rs117482282	Chr6:165511471	NR	European	None	1843	NR	3.08	[1.991-4.762]	4.00E-07	RPL17P25	[27]
4	rs12629668	Chr3:147209179	NR	European	None	1843	NR	1.40	[1.228-1.591]	4.00E-07	ZIC1	[27]
5	rs1149833	Chr13:50750876	NR	European	None	1843	NR	1.34	[1.19-1.5]	5.00E-07	DLEU1	[27]
6	rs1406230	Chr2:29583321	NR	European	None	1843	NR	1.44	[1.25-1.67]	5.00E-07	ALK	[27]
7	rs11706588	Chr3:126448513	C	European	None	1843	0.1305	1.89	[1.64-2.14]	7.00E-07	CHCHD6	[27]
8	rs140306040	Chr7:62321151	NR	European	None	1843	NR	1.96	[1.5-2.57]	7.00E-07	Unknown	[27]
9	rs35498131	Chr16:9120809	NR	European	None	1843	NR	1.79	[1.42-2.25]	7.00E-07	USP7, C16orf72	[27]
10	rs34954281	Chr2:152225877	NR	European	None	1843	NR	1.48	[1.27-1.74]	9.00E-07	TNFAIP6	[27]
11	rs1990145	Chr2:75877650	A	Australians (European)	None	881	0.268	2.02	[1.50-2.72]	4.00E-06	Unknown	[24]
12	rs4771506	Chr13:106496000	C	Australians (European)	None	881	0.263	1.97	[1.46-2.64]	7.00E-06	LINC00343	[24]

Chr, chromosome; CI, confidence interval; EAF, effect allele frequency; NR, not reported; OR, odds ratio; SNP, single nucleotide polymorphism

Table S6. Quality assessments of included case-control studies

Author (Year of Publication)	Newcastle - Ottawa Quality Assessment Scale for Case-Control Studies									
	Selection				Comparability	Exposure			Total stars	Reference
	1	2	3	4	1	1	2	3		
Buraczynska M (2011)	a	a	b	a	Models adjusted for age, sex, blood pressure, BMI, diabetes duration, and HbA1C	a	a	n/a	7	[66]
Ciccacci C (2013)	a	a	b	a	Control matched by age	a	a	n/a	7	[62]
Luo J (2013)	a	a	b	a	Models adjusted for BMI	a	a	n/a	7	[63]
McAuley AK (2014)	a	a	b	a	Models adjusted for age, sex, duration of diabetes, and hematological levels of HbA1c, fasting glucose, and serum creatinine	a	a	n/a	7	[60]
Hosseini SM (2015)	a	a	a	a	Models adjusted for age, gender, diabetes duration and mean HbA1C	a	a	n/a	7	[61]
Cheung CY (2016)	a	a	b	a	Models adjusted for age, sex, duration of diabetes, the presence of HT, and HbA1C	a	a	n/a	7	[59]
Azzam SK (2019)	a	a	b	b	Models adjusted for age, diabetes duration, hypertension and dyslipidemia, and smoking	a	a	n/a	6	[58]
Shawki HA (2020)	a	a	b	a	Control matched by age and gender; models adjusted for age, diabetic duration, gender, HbA1 c, creatinine and lipid profile	a	a	n/a	7	[65]
Magazova A (2022)	a	a	b	a	Models adjusted for age, gender, diabetes duration	a	a	n/a	7	[64]

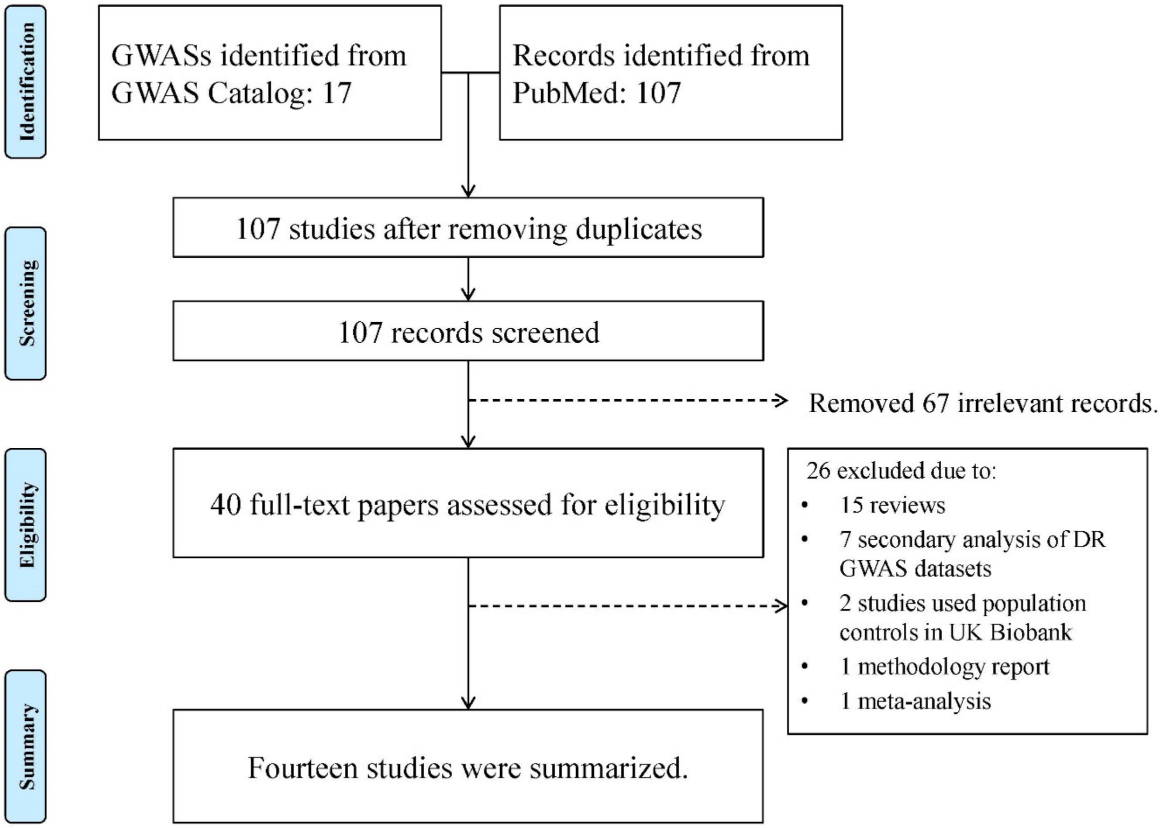

Figure S1. GWAS catalog and literature search and results of literature review

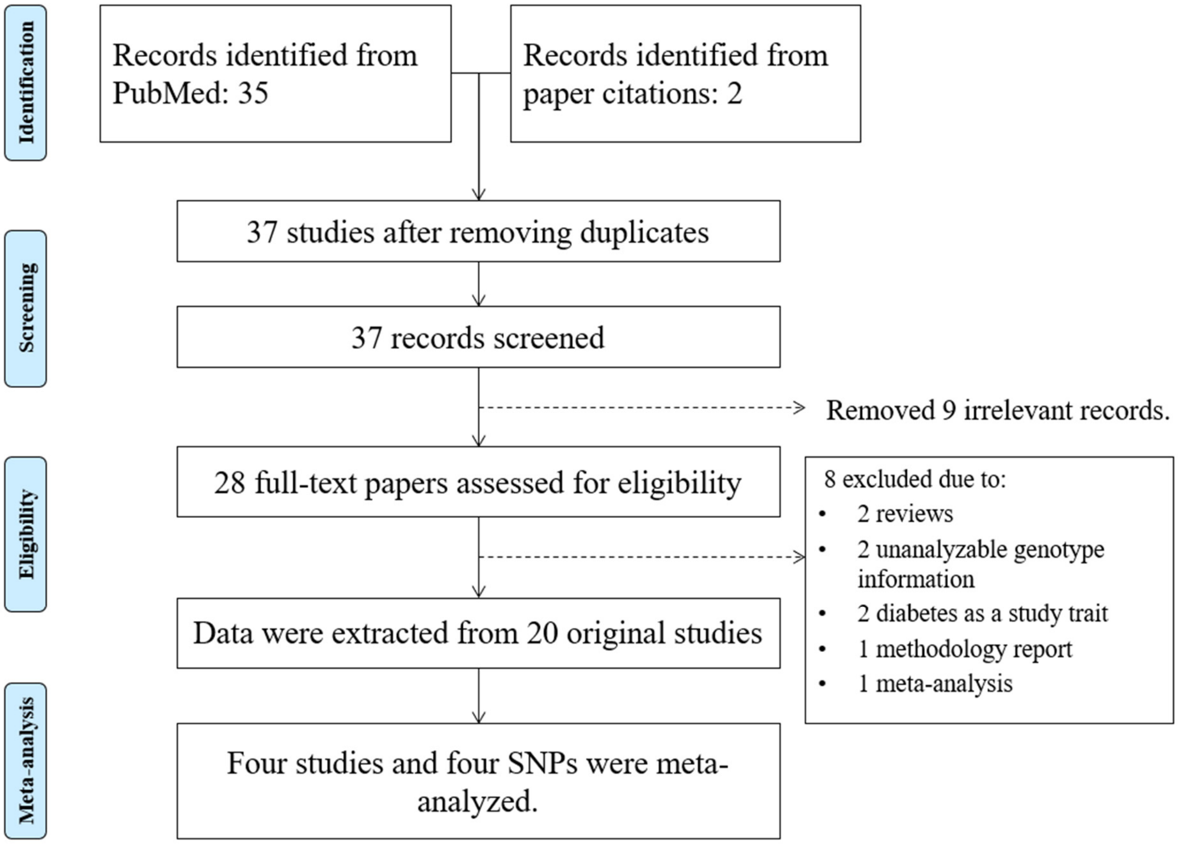

Figure S2. Literature search for replication studies of top GWAS signals

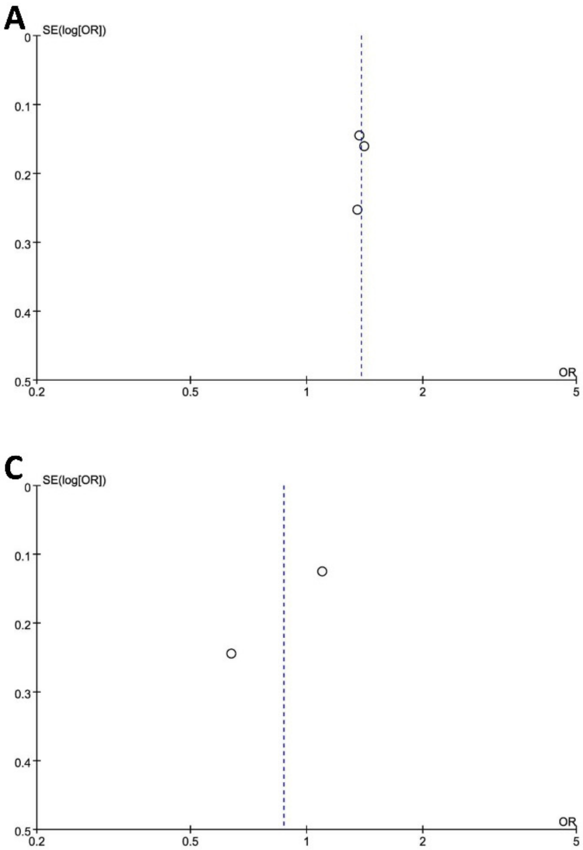

Figure S3. Funnel plots of studies outcomes used in meta-analysis Study outcomes used in each meta-analysis did not show significant deviation in Funnel plots suggesting a low odds of introducing publication bias. A. rs4462262 (effect allele T) *ZWINT-MRPS35P3*. B. rs12219125 (T) *PLXDC2-NEBL*. C. rs4838605 (C) *ARHGAP22*. D. rs17376456 (G) C5orf36.

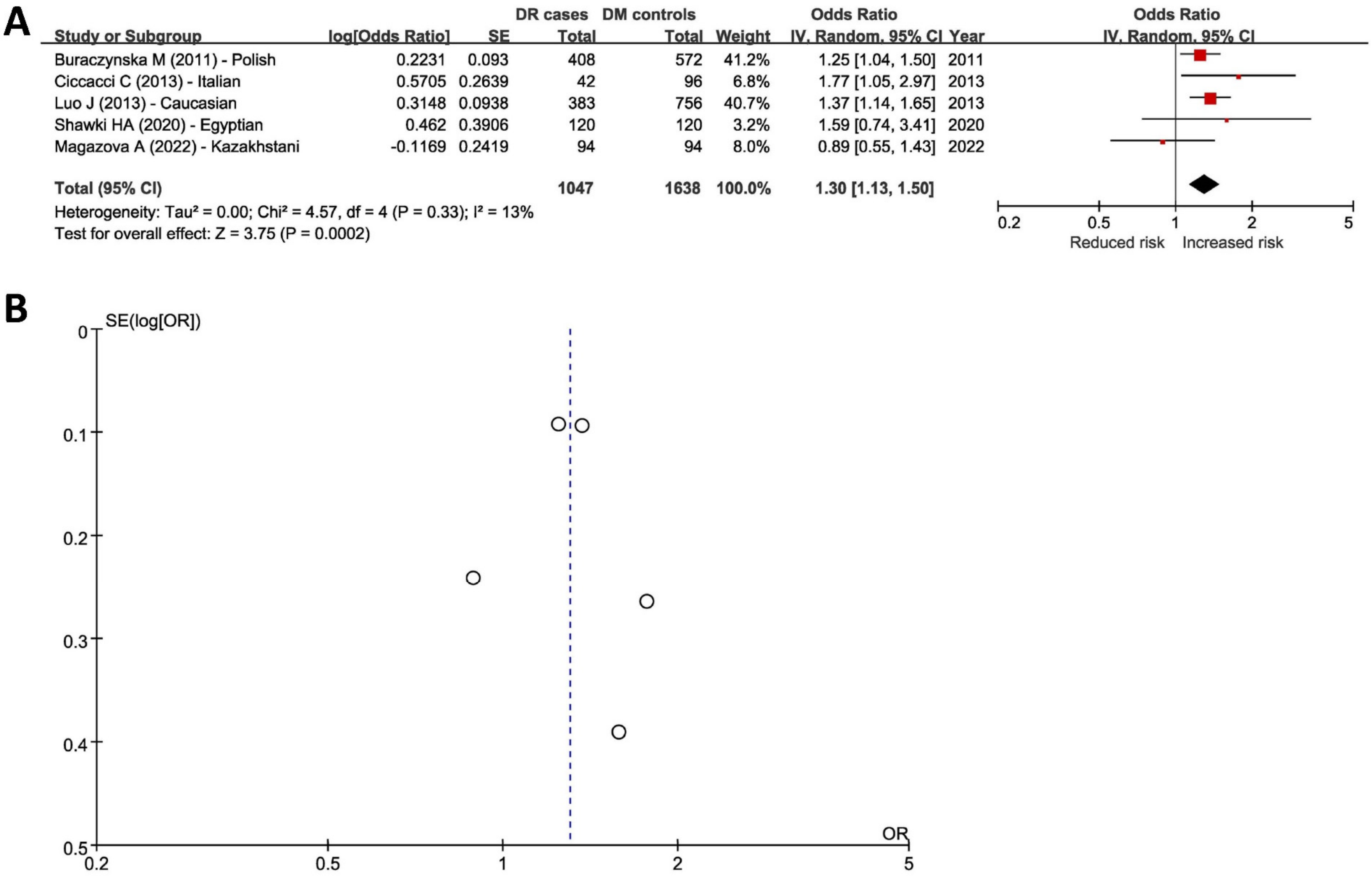

Figure S4. Meta-analysis of genetic association of rs7903146 (T, *TCF7L2*) with diabetic retinopathy A. Combined odds ratio suggested a significant association of rs7903146 T allele with increased risk of diabetic retinopathy. Heterogeneity between included studies was low as shown by I^2. B. Study outcomes did not show significant deviation in Funnel plots suggesting a low odds of publication bias.
